# Transmission of Microcystins in Natural Systems and Resource Processes: A Review of Potential Risks to Humans Health

**DOI:** 10.3390/toxins15070448

**Published:** 2023-07-06

**Authors:** Xueli Ren, Yuting Wang, Kenian Zhang, Yi Ding, Wanqing Zhang, Mengyi Wu, Beiqi Xiao, Peng Gu

**Affiliations:** School of Environment and Civil Engineering, Jiangnan University, Wuxi 214122, China; renxueli@jiangnan.edu.cn (X.R.); 6211405029@stu.jiangnan.edu.cn (Y.W.); 1103220201@stu.jiangnan.edu.cn (K.Z.); 6221402065@stu.jiangnan.edu.cn (Y.D.); 6221402050@stu.jiangnan.edu.cn (W.Z.); 6221402037@stu.jiangnan.edu.cn (M.W.); 6221402038@stu.jiangnan.edu.cn (B.X.)

**Keywords:** microcystins, aquatic ecosystem, resource utilization, agricultural plants

## Abstract

The rapid rise of microcystins (MCs) poses a serious threat to global freshwater ecosystems and has become an important issue of global public health. MCs have considerable stability and are the most widely distributed hepatotoxins. It cannot only accumulate in aquatic organisms and transfer to higher nutrients and levels, but also be degraded or transferred during the resource utilization of cyanobacteria. No matter which enrichment method, it will lead to the risk of human exposure. This review summarizes the research status of MCs, and introduces the distribution of MCs in different components of aquatic ecosystems. The distribution of MCs in different aquatic organisms was summarized, and the potential risks of MCs in the environment to human safety were summarized. MCs have polluted all areas of aquatic ecosystems. In order to protect human life from the health threats caused by MCs, this paper also proposes some future research directions to promote MCs control and reduce human exposure to MCs.

## 1. Introduction

The increase in lake nutrient load and the frequent occurrence of cyanobacterial blooms caused by climate change are a major problem facing aquatic ecosystems at present [1]. Many of the algae genera such as *Microcystis*, *Anabaena*, and Phytoplankton produce a large amount of secondary metabolites—microcystins (MCs) [2]. MCs are a kind of bioactive heptapeptide monocyclic hepatotoxin. They will cause different degrees of damage to various organs of the human body while endangering water quality. There are cyclic structures and spacer double bonds in its structure [3]. Therefore, it is considerably stable, can resist external physical and chemical effects, and is hard to degrade under natural conditions [4]. Among them, the most commonly detected MCs in natural water are MC-LR, MC-RR, and MC-YR. MC-LR is the most toxic [5]. Long-term frequent exposure can cause liver toxins, kidney toxins, neurotoxins and other bioactive compounds that damage the human body [6]. Therefore, it is urgent to explore the transformation of MCs in water and the risk assessment of human health.

MCs accumulate in aquatic organisms and are transmitted to higher nutritional levels as the trophic level of the aquatic ecological food web in the ecosystem increases, which will have a variety of adverse effects on phytoplankton, zooplankton, fish and even humans [7]. Among them, zooplankton is the most important link in the spread of MCs in the food web [8]. It can transfer the substances and energy produced by primary producers to fish in freshwater ecosystems, and can also enrich and transfer the toxin to other organisms in the food web as the carrier of MCs in the aquatic food web [9]. Fish can accumulate MCs in two ways: the first is eating cyanobacterial cells or other cyanobacterial toxin-contaminated organisms, and zooplankton is a major part of the fish diet. The second is the direct absorption of dissolved MCs from the surrounding water [10]. Human health is often associated with consistent exposure to low concentrations of MCs [11]. In daily life, humans can be exposed to MCs in the following ways: physical contact; drinking contaminated water; eating contaminated food; edible algae dietary supplements and hemodialysis, and these types of contact generally interweave. Among them, drinking water contaminated with MCs and eating contaminated food are the most common routes of exposure [12]. Therefore, it is necessary to further explore the migration and transmission of MCs, reduce the concentration of MCs that humans may be exposed to, and maintain the MCs standard of 1 μg L^−1^ in drinking water stipulated by the World Health Organization (WHO) to protect human health [13].

MCs can also be migrated and enriched through the resource utilization of cyanobacteria, which has adverse effects on animals and humans [9]. At present, various methods are used in the resource treatment of cyanobacteria including utilizing the abundant organic matter of cyanobacteria to compost, using rich proteins of cyanobacteria as feed raw materials, and producing hydrogen with the use of photosynthesis in cyanobacteria as a new biomass energy [14]. Among them, the method that mixes cyanobacteria with fodder to feed black soldier flies has become a novel application direction of cyanobacteria resource utilization. Through the mixture of cyanobacteria and feed in different proportions, the effect of maximum utilization of cyanobacteria by black soldier fly can achieve, and harmful cyanobacteria are treated meanwhile [15]. The black soldier fly can be used to feed pigs, chickens and other animals, and can also be added as an antimicrobial peptide to fertilizer to optimize composting efficiency and refine biodiesel [16]. It will inevitably migrate and enrich the MCs in cyanobacterial cells to the human body, and accumulate in various organs of the human body. When the accumulation reaches a certain value, it will lead to skin allergy, acute gastroenteritis, liver dysfunction and even death [17].

At present, more studies have reported the harm of MCs in water and its influence on human health, while the relationship between the migration and enrichment of MCs and the risk assessment of human health is lacking, which needs deeper summary and discussion [18]. Through recent data analysis, this review comprehensively discusses the migration and enrichment of MCs in natural ecosystems and the process of resource utilization of cyanobacteria, and evaluates the human health risks exposed to MCs. The aim is to provide control and utilization of MCs in the future, understand the metabolism and bioavailability of these toxins in exposed organisms, and reduce their threat to human work and life.

## 2. Exposure Pathways of MCs in Aquatic Food Chain

**Figure 1:**
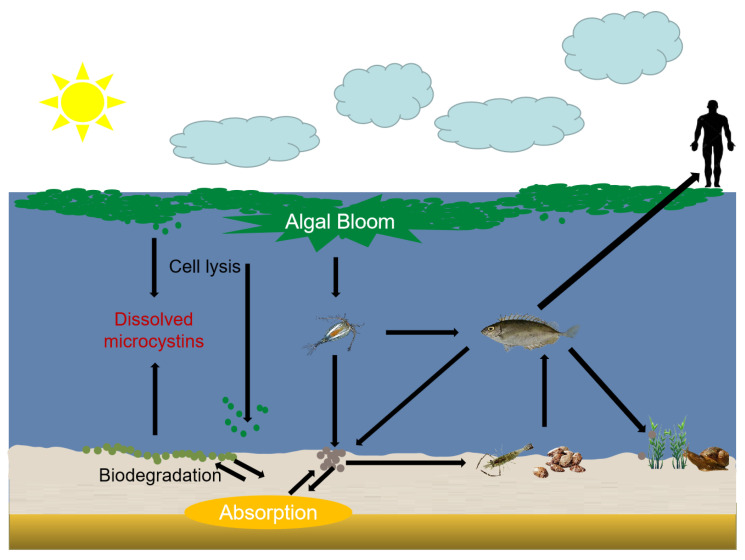
Bioaccumulation and transfer of microcystins (MCs) in aquatic ecosystems: a schematic diagram (Modified from (Song et al., 2015)).

In the natural aquatic ecosystem, cyanobacteria enter the decline period after the four stages of overwintering, recovery, growth, floating, and release a large number of secondary metabolites [18]. The effects of MCs on the water environment, animal predation and human health should not be ignored [19]. The aquatic food web will enrich and migrate MCs to phytoplankton, zooplankton, benthic herbivores, fish, and humans (Figure 1). MCs are considered to have adverse effects on aquatic species [20]. The toxic mechanism of MCs on experimental animals has been verified in many studies, including histological, biochemical and behavioral effects [9]. Studies have shown that the immune indexes, blood cells and cell enzyme activities of fish have changed significantly after exposure to purified MCs or crude extracts of cyanobacterial [7]. Therefore, the accumulation and migration mechanism of MCs in aquatic organisms is necessary to study the harm of MCs to the environment [21].

### 2.1. Accumulation of Zooplankton

Different species of zooplankton can coexist with cyanobacteria in water, and most zooplankton accumulate MCs in their own bodies [22]. According to the literature, the concentration of MCs in zooplankton is the highest, reaching 1300 μg g^−1^ dry weight (dw), with an average of about 383 μg g^−1^ dw. Among them, herbivorous zooplankton, such as rotifers and copepods, are less sensitive to cyanobacterial bacteria, while copepod dinoflagellates are more sensitive to cyanobacterial toxins than water fleas [2]. There are many studies to measure the accumulation of MCs in zooplankton by natural conditions [8]. Among them, Sotton et al. found that *Daphnia*, *Bosmina* and *Chaoborus* larvae can achieve higher MCs accumulation by studying the nutritional transfer of MCs between zooplankton groups and invertebrates at different depths in Lake Harville (Switzerland) [23], and the concentration of MCs in zooplankton generally does not change with the change of water depth. The concentration of MCs in zooplankton at dusk was significantly higher than that at other time periods, indicating that the accumulation of MCs were related to time. Moreover, research found that zooplankton and fish feeding on zooplankton will have a biomagnification phenomenon, which is gradually enriched in organisms through the increase in trophic level, resulting in an increasing concentration of MCs [23]. The sensitivity of different zooplankton to MCs still needs further experiments.

### 2.2. Accumulation of Benthic Herbivores

Benthic herbivores are mostly foraging animals in the lower layer of water, including invertebrates such as shellfish, crabs, freshwater lobsters, sea anemones, starfish, snails, sand silkworms, and sea cucumbers [24]. Studies have shown that benthic herbivores can accumulate MCs in the waters where cyanobacteria blooms break out and transfer along the food chain, and the accumulation pattern of MCs in benthic herbivores depends on their species, seasons, metabolism and the purification rate of MCs [24]. Among them, the MCs’ concentrations of *Unio douglasiae* and *Mytilus edulis* were as high as 420 μg g^−1^ dw and 336.9 μg g^−1^ dw, respectively. Studies have showed that the Asian clam mainly feeds on *Microcystis* and continuously accumulates MCs in the body [25]. After exposure to cyanobacteria blooms for a period of time, the rate of degradation of MCs by Asian clams decreased, resulting in the purification of toxins in the body, especially hepatotoxins. The highest concentration of accumulated MCs were as high as 4000 ng L^−1^ dw [26]. Eastern oyster could also purify *Microcystis* in water, but eastern oyster could not degrade MCs in the body itself. The accumulation of MCs were higher than that of Asian clams, and the highest concentration of MCs was as high as 5000 ng L^−1^ dw [27]. Therefore, the toxicity of MCs in eastern oysters was much greater than that in Asian clams. In October, the highest concentration of MCs were observed in the hepatopancreas and intestine of *Sinotaia histrica* [28]. High concentration of MCs into bivalves will inhibit their immune function, resulting in a decline in physiological function [29]. Meanwhile MCs can reduce activity of glutathione S-transferases (GSTs), destroy the detoxification mechanism of MCs, and MCs achieve residual in vivo. These most important resource bivalves are easy to gather in the lower reaches of the estuary, and are directly consumed by benthic carnivores or humans, and then further transmit MCs through the food web [30].

### 2.3. Accumulation of Gastropod

In addition to the zooplankton and bivalves mentioned above, gastropods are also an important link between primary producers and senior consumers, and often play a key role in the construction of aquatic communities [31]. Recent studies have shown that gastropods such as *Lymnaea stagnalis*, *Physa gyrina*, and *Heliosoma trivolvis* are often exposed to cyanobacterial waters. The concentration of MCs in their tissues is proportional to the content of phytoplankton that contain MCs in the body. They accumulate toxins by preying on phytoplankton that contain MCs and absorb soluble MCs. The concentration can reach 40–140 μg g^−1^ dw. Further research has found that MCs can accumulate in different organs of gastropods [32]. Taking Taihu Lake, the third largest freshwater lake in China, as an example, the freshwater snail feeds on *Microcystis aeruginosa* in the lake, and the accumulation of MCs in hepatopancreas is the largest, followed by digestive tract and gonad. After accumulation of MCs for 5 weeks, freshwater snail will begin to purify its own toxins in a period of three weeks which can remove about 65% of MCs in the body [33]. The remaining MCs can be accumulated in gastropods and transmitted to senior consumers.

### 2.4. Accumulation of Fish

A large number of studies have shown that fish, as an senior consumer of the aquatic biological chain, accumulate MCs only second to zooplankton, up to an 874 μg g^−1^ dw maximum [8]. According to the transmission of the food chain, fish mainly prey on zooplankton or benthic animals. In the waters where cyanobacteria blooms break out, zooplankton and benthic animals will enrich MCs and then be predated by fish. Meanwhile, fish can also absorb dissolved MCs directly from the surrounding water to achieve the accumulation of MCs in different organs of fish [22]. MCs can produce toxic mechanisms in fish through histological, biochemical and behavioral effects. Once fish absorb MCs into the body, the circulation of blood will quickly transport the toxins to various organs or tissues [34]. In the intestines of fish, the concentration of MCs accumulated by phytoplankton was the highest, followed by carnivorous fish and omnivorous fish. The accumulation of MCs in the liver, kidney and muscle of carnivorous fish and omnivorous fish was generally higher than that of herbivorous fish [35]. In omnivorous fish such as tilapia, the average content of MCs accumulated in the liver was 0.15 μg g^−1^ dw, while only 0.012 μg g^−1^ dw was accumulated in the muscle [36]. The above phenomena indicate that fish do enrich MCs in different organs in their bodies through the food chain.

At present, the accumulation of MCs have been found in organs, including fish liver, intestine, kidney, gallbladder, gill, muscle and brain. In addition to directly attacking fish liver organs, MCs can adversely affect immune systems [37]. A number of studies have shown that fish exposure to MCs can lead to significant changes in immune indicators, blood cells and cell enzyme activities: histological damage to fish kidneys, hearts, gills and other organs, and even organ failure [38]. At the same time, it will affect the normal development process of fish, resulting in the abnormal development of juvenile fish, such as skeletal deformity, head shrinkage, body bending, heart enlargement and so on [39]. The chronic toxicity caused by the continuous exposure of MCs will cause the imbalance of osmotic regulation in the fish body and increase the volume of liquid in the intestine, resulting in the inability of the fish body to digest the excess water in the body, the swelling of the body, the inability to survive, or the destruction of the osmotic pressure of the fish body [40]. The water in the body will continue to seep out from the gills and surface of body, the fish lose plenty of water and die eventually. In addition to the above effects on organs and body shape, it also causes irreversible damage to enzyme activity and DNA of fish. After exposure to MCs for a period of time, lipid peroxidation occurs in the fish body, and the activities of antioxidant enzymes such as superoxide dismutase (SOD), peroxidase (POD) and catalase (CAT) are greatly reduced [40]. These antioxidant enzymes are the basis for responding to oxidative damage, and self-purification in the body is carried out by eliminating the accumulated MCs, and the decrease in enzyme activity eventually leads to the fish body being unable to resist the effects of strong oxidation on organs and tissues. Moreover, the fish body catalyzes the coupling of MCs and glutathione (GSH) through detoxification enzymes such as GSTs. After coupling, the toxicity of MCs is greatly reduced, and it can accelerate the excretion of MCs, which is conducive to resisting the damage caused by MCs. Such detoxification mechanisms are usually carried out 15 days after fish are exposed to MCs [41]. If fish are exposed to excessive MCs, the activity of such enzymes will be affected, resulting in the stagnation of detoxification mechanisms. For example, in zebrafish, fish eggs, etc., after 24 h of exposure to MCs, GSTs enzyme activity is greatly reduced, resulting in the inability to expel MCs from the body through coupling with bile. In addition to the above GSH detoxification mechanism, protein phosphatases (PP) 1 and PP2A are also widely considered to be the main mechanisms of MCs toxicity. However, PP1 and PP2A cannot resist MCs beyond the tolerance threshold, so the cells are hyperphosphorylated, leading to apoptosis [42].

In summary, after fish directly absorb MCs in water or eat phytoplankton, zooplankton and benthic herbivores containing MCs, their hepatopancreas, heart, kidney and intestine are directly damaged [22]. When the concentration of MCs exceeds the tolerance threshold of organs in fish, the decrease in enzyme activity in vivo leads to the inability of detoxification mechanisms, which leads to the excessive accumulation of MCs, and then transmits through the food web [7].

## 3. Exposure Pathways of MCs in the Process of Resource Utilization

At present, the deterioration of water quality and water odor caused by the outbreak of cyanobacterial blooms affect the normal work and life of citizens. Chemical algicides such as copper ion preparations and erythromycin thiocyanate play an important role in eliminating cyanobacterial blooms [43]. Although the effect is immediate, it will cause secondary pollution of heavy metals and antibiotics. Biological methods include microbial control, growing aquatic plants, etc. The algae control is safe and effective; however, it is hard to work in a short period of time. Salvage is currently the most direct and effective measure to reduce the ecological disaster of cyanobacteria water pollution and reduce the intensity of outbreak again [44]. Taking Taihu Lake as an example, when the cyanobacteria bloom broke out in 2007, about 1000 tons of cyanobacteria were salvaged every day, but cyanobacteria could not be eaten directly by poultry and livestock. A large number of salvaged cyanobacteria could not be treated in time and effectively. Long-term accumulation was easy to rot and stink, releasing harmful gases such as hydrogen sulfide. A large number of algal toxins, heavy metals and other harmful substances will cause secondary pollution to the environment [45]. Cyanobacteria are rich in nutrients, and the prospect of resource utilization is broad. There are several ways to utilize cyanobacteria at home and abroad: utilizing cyanobacteria to prepare cyanobacteria compost and cyanobacteria biogas manure by abundant organic matter, nitrogen, phosphorus and potassium in cyanobacteria; utilizing the rich protein in cyanobacteria as feed raw materials; utilizing the light and hydrogen of blue-green algae as a new type of biomass energy; the rich nutrients in cyanobacteria can be used to cultivate microbial materials [46]. In the process of resource utilization of cyanobacteria, the exploration of the migration and accumulation mechanism of MCs is particularly important.

### 3.1. Cyanobacteria Compost

Direct application of cyanobacteria as fertilizer to the field will lead to a large amount of MCs and heavy metal residues, which seriously endangers the growth of crops and human health [20]. At the same time, according to research, when soil containing MCs were used to grow green vegetables, the growth rate of green vegetables was significantly inhibited, and the content of MCs absorbed by the above-ground part of green vegetables increased with the increase in the content of MCs exposed to the ground [47]. The non-degradable MCs will accumulate in the soil for a long time, endangering human health. In recent years, many studies have composted salvaged cyanobacteria. Sasaki, Yoshikuni directly added salvaged and dried cyanobacteria powder to soil to study the fertilizer effect on crops [48]. The algae powder is a kind of organic material that is easy to dissolve and mineralize. It slowly dissolves in the soil and releases CO_2_, N and P. At the same time, it improves the activity of soil microbial enzymes and promotes the growth of lettuce. Asghari, Zeinalzadeh, Kheirfam, Habibzadeh Azar studied the effect of cyanobacteria composting [49]. During the composting process, the plant germination index increased rapidly to 75%, and it was found that the fertilizer with nitrogen fixing agent had a better plant growth effect. It can be seen that the use of high temperature composting treatment technology can be more efficient for the resource utilization of cyanobacteria. At the same time, most MCs can be degraded after composting. When the water content was adjusted to 55% and the C/N ratio was 25, the degradation rate of MCs could reach more than 90%. The intake of MCs in vegetables cultivated by cyanobacteria compost was much lower than the WHO’s allowable daily intake of 0.04 μg kg^−1^ d^−1^ [50].

### 3.2. Cyanobacteria Biogas Fertilizer

The most common method of resource utilization of cyanobacteria is to make high-quality organic fertilizers from anaerobic fermentation of cyanobacteria [51]. At present, the technology of biogas fertilizer production from cyanobacteria has become mature, and the current research hotspot has focused on the non-toxic and harmless environmental protection application of cyanobacteria biogas fertilizer. The study found that the salvaged Taihu cyanobacteria were inoculated with an appropriate amount of activated sludge and then anaerobically fermented [52]. Compared with the methane content produced by the fermentation of livestock and poultry manure, the fermentation of cyanobacteria would produce higher methane [53]. At the same time, the biogas slurry residues were rich in nutrients such as nitrogen, phosphorus, potassium and amino acids, which can be used as high-quality organic fertilizer to cultivate crops. During the whole process of biogas fertilizer production, it was found that the degradation rate of MCs in an anaerobic state was much higher than that in natural storage [54]. Ma H et al. found that the MCs were almost completely degraded after the cyanobacteria recovered in Dianchi were inoculated with an appropriate amount of activated sludge and subjected to anaerobic fermentation [55]. At the same time, Aravind et al. mixed cyanobacteria mud with composite environmental induction material ECO-U100 in a ratio of 100:2 to obtain a non-toxic and pollution-free environmental organic fertilizer [56], which can significantly improve rice quality compared with chemical fertilizer. Therefore, in the process of resource utilization of cyanobacteria biogas fertilizer, MCs can be well degraded and will not cause such harm to the environment [57]. However, in this process, it is necessary to pay attention to the production of other harmful gases in the biogas production process, such as hydrogen sulfide (H_2_S), methyl mercaptan (CH_3_SH) and methyl sulfide (C_2_H_6_S), and the purity of anaerobic biogas fermentation needs to be improved by desulfurization [58]. At present, the utilization of cyanobacteria has made great progress abroad and has been put into use initially. However, it is still in the preliminary stage in China. It is necessary to further solve the problem of harmful gases in the process of resource utilization, so that cyanobacteria can be used as organic fertilizer to alleviate the shortage of resources [59].

### 3.3. Raw Material for Feeds

The utilization of cyanobacteria resources as feed is a means with broad application prospects [60]. Nagarajan et al. found that the specific growth rate of tilapia after long-term consumption of feed containing cyanobacteria powder was higher than that of the control group [61]. At the same time, adding a certain amount of cyanobacteria powder to the feed can increase the intake of fish. If the concentration of MCs in cyanobacteria powder can be reduced, it has great value prospects to use cyanobacteria powder as a fish feed protein source [62]. The impact of MCs should be considered when using cyanobacteria as feed, because the feed carries a large number of MCs, and the ingested MCs enter the food chain to accumulate and purify in aquatic organisms [2]. As a zooplankton-eating fish, tilapia is active in the middle and lower reaches of the food chain. After eating the feed containing cyanobacteria, most of the MCs can reduce the toxins in the fish by excretion [63]. The remaining MCs are digested by tilapia and enter the kidney, heart, muscle and other tissues of the fish to accumulate and transmit to the upper food chain [64]. According to the detection of MCs content in muscle tissue of fish with different nutritional levels on the food chain of Chaohu Lake in Anhui Province, it was found that MCs in the upper trophic layer of the food chain, such as carnivorous and omnivorous fish, were significantly higher than those in phytoplankton-eating fish and herbivorous fish downstream of the food chain [65]. This shows that when cyanobacteria are used as feed, they can be used by primary consumers to migrate through the food chain, which in turn threatens human safety [66].

## 4. The Impact of Algal Toxins on Human Health and the Assessment of Human Health Risks of Cyanobacteria Recycling

Eutrophication of freshwater bodies is becoming increasingly serious in the world [67]. When cyanobacteria blooms occur in large lakes or rivers, MCs in cyanobacteria will migrate and transfer with the above-mentioned natural ecosystems and resource utilization processes, and eventually accumulate in humans, which will have adverse effects on human health [68]. When MCs are exposed and absorbed by blood, they will be transported to various organs and distributed to liver, brain, kidney, lung, heart, reproductive system [69]. With the accumulation of concentration, it will produce chronic poisoning, leading to nausea, dizziness, vomiting and other effects [70]. When MCs accumulate in organs to a certain concentration, it will promote the formation and spread of cancer cells. The effect of MCs on human health is related to the concentration of MCs in drinking water [71]. Most of the studies of MCs on organs are proved by human cell and animal experiments in the laboratory [72].

### 4.1. The Harm of MCs to Liver

The liver contains the most organic anion transporting polypeptides (OATPs) required to assist MCs to enter cells, so it is the main target organ of MCs. At the same time, MCs can also enter the liver through the bile acid transport system [69]. Many studies have shown that MC-LR can increase the liver weight and lead to significant histopathological changes, including liver parenchymal structural damage, hepatocyte swelling, vacuolar malnutrition, loss of cell membrane integrity, necrosis, steatosis and lymphoid center hyperplasia, and inflammatory response [73]. Palikova et al. found that the content of free MC-LR in the serum of mice fed with MC-LR was less than that in liver [74], indicating that MC-LR mainly accumulated in the liver. It was found for the first time that MC-LR caused cholestasis in fish. Liver mitochondria edema occurred at day 1, and the damage to mitochondria was the most serious at day 2. However, after 7 days of exposure to MC-LR, it was found that the damage to liver mitochondria recovered to some extent. This may be because the continuous accumulation of MC-LR in the body gradually caused damage to the liver. With the decomposition of the toxin and the initiation of the toxin clearance mechanism in the body, the recovery of the liver was promoted, so that the structure of mitochondria gradually returned to normal. Long-term exposure to MCs will lead to the failure of the detoxification mechanism of organisms, thus becoming unable to carry out normal MCs metabolism. In summary, MC-LR causes a greater damage to the liver of animals and even humans [75]. Long-term intake will lead to liver cancer lesions. Therefore, it is urgent to study the migration pathway of MCs to control the entry of MCs into the human body [76].

### 4.2. The Harm of MCs to Kidney

Kidney is another important target organ of MCs, which plays an important role in MCs metabolism [77]. MCs are transported to renal tubular cells through a similar transport mechanism to the liver, causing kidney damage. In the laboratory, intraperitoneal injection of MCs in mice showed that tritium-labeled derivatives were highly accumulated in the liver and also distributed in the kidney [78]. When the mice were intraperitoneally injected with 10 μg kg^−1^ of MC-LR for eight weeks, the glomerular collapse and basement membrane thickening were caused, indicating that MC-LR caused significant renal damage in mice. In recent years, although the treatment of cyanobacteria in Taihu Lake has achieved certain results, cyanobacteria bloom pollution still occurs frequently in the summer [79]. Among them, silver carp and bighead carp, two kinds of zooplankton-eating fish, were exposed to MCs for a long time. There were renal tubular edema, mitochondrial deformation and other phenomena. It was found that MCs caused glomerular cell necrosis, lysosomal hyperplasia, nuclear condensation and degenerative lesions [80]. The accumulation of high concentrations of MCs in the kidney promotes the ultrastructural pathological changes of the renal membrane structure, which affects the regulation of hormones, and leads to renal insufficiency [81]. Therefore, according to a large number of studies, MCs can cause kidney cell damage in mammals.

### 4.3. The Harm of MCs to Nervous System

MCs are transported to the brain tissue through the blood–brain barrier through the organic anion transporting polypeptides (OATPs) for distribution and accumulation, which will cause abnormal development of the nervous system in the human body, causing neurotoxicity, dizziness, nausea, vomiting, lethargy, visual impairment, tinnitus, epilepsy and a series of neurotoxicity symptoms [82]. According to a series of proteomics studies, MCs can damage the development and function of the nervous system by affecting the cytoskeleton, oxidative stress and energy metabolism, and inhibiting protein phosphatase in brain tissue [83]. After feeding 5-day-old mice with an MCs extract, Diez-Quijada et al. studied the function of OATPs in transporting MCs using the mouse whole brain cell system [84], and found that MC-LF and MC-LW have stronger potential to induce neurotoxicity in animals than MC-LR. However, in the study of rat brain tissue, it was found that the accumulation of MC-RR was higher than that of other MCs, suggesting that the blood–brain barrier of rats selectively inhibited highly toxic MCs. In the famous Brazilian renal dialysis event, 89% of patients with MCs poisoning had neurological symptoms such as visual impairment and mild deafness [6]. The above facts show that MCs have a great adverse effect on the development and function of the nervous system, which can cause related neurodegenerative diseases such as Alzheimer’s disease and Parkinson’s disease.

### 4.4. Other Toxicity of MCs

In addition to causing different degrees of damage to the liver, kidney and nervous system, MCs can also cause reproductive toxicity, immune toxicity and genetic toxicity [85]. A study has found that male rats had testicular damage, sperm motility and survival and development ability were greatly reduced after injection of MCs crude extract [34], and sperm quality was reduced. After 30 days of a low-dose MCs exposure test, female medaka were found to have atrophy of gonadal tissue and a decrease in yolk content, which had a great negative impact on the ovary. MCs can cause harm to the body by damaging genetic materials such as DNA, chromosomes and genes. At present, the study of immunotoxicity mainly focuses on its effect on immune cell nuclear immune molecules. Animal experiments have shown that MCs have a significant inhibitory effect on multiple aspects and levels of the mouse immune system [86]. MC-LR can reduce the killing activity of mouse natural killer (NK) cells against mouse lymphoma cell YAC-1 cells, resulting in a decrease in the ability of mice to resist lymphoma, showing a certain degree of immune damage [87]. At the same time, it can also reduce the nitric oxide (NO) production of macrophages, resulting in the destruction of the immunopathological process of killing microorganisms.

**Figure 2:**
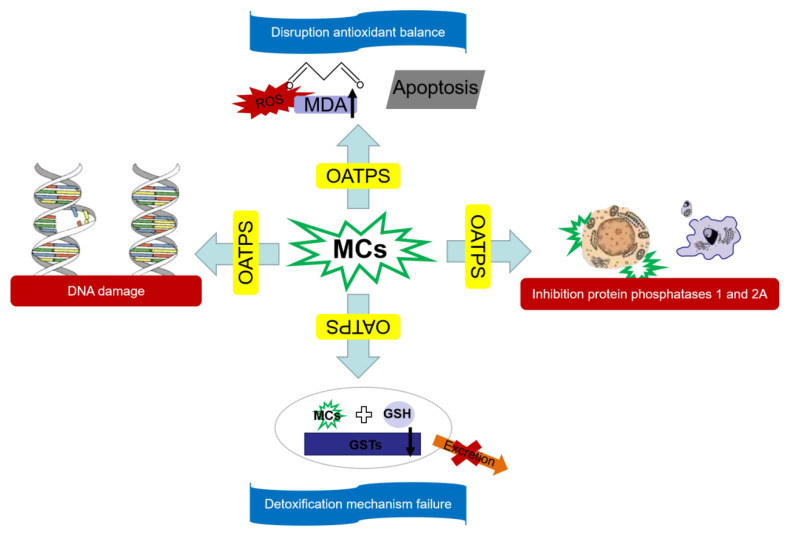
Four toxic mechanisms of microcystins (MCs) on human body.

The toxic effects of MCs on humans may have the following four mechanisms (Figure 2): First, MCs cannot pass through the cell membrane by passive transport [88]. In most cases, they are transported by OATPs carriers. OATPs is distributed in different contents in organs and tissues of humans and mammals. There are different expressions in liver, kidney, intestine, heart and gonads, so MCs will selectively attack these organs [89]. Secondly, MCs inhibit protein phosphatase activity. After MCs enter cells, MeAsp residues first inhibit the activity of PP1 and PP2A, and then the Mdha group of MCs covalently binds to the cystine serine residues of PP1 and PP2A, resulting in irreversible damage to PP1 and PP2A [90]. Finally, the phosphorylation and dephosphorylation of various proteins in the cell are unbalanced, and the biochemical process is disordered, resulting in cell damage. Furthermore, MCs can cause oxidative imbalance in cells, and MCs can increase MDA content in cells, resulting in lipid peroxidation in cells, causing the failure of the body’s antioxidant defense system and abnormal apoptosis of cells [91]. Finally, MCs can induce DNA mutations, damage DNA structure, and inhibit DNA repair. MCs directly affect the morphology of the nucleus. MCs-induced ROS also act on DNA structure and induce chromosome breakage [92]. When the above toxicity mechanism occurs, detoxification enzymes such as GSTs will catalyze the coupling of MCs with glutathione (GSH). The toxicity of the coupled MCs is greatly reduced and can be accelerated in the body, which is the detoxification mechanism of the human body to cope with the harm of MCs. However, when the human body is exposed to low-dose MCs for a long time, oxidative damage leads to a decrease in the content of GSTs detoxification enzymes and GSH, MCs cannot be excreted from the body, and the detoxification mechanism fails [22].

### 4.5. Conclusions and Outlooks

This study combines the distribution of MCs in aquatic organisms and their exposure pathways in the process of resource utilization of cyanobacteria to reveal the enrichment pathway of MCs. It enriches the lack of resource utilization of MCs in the field of aquatic toxins.

The large-scale outbreak of cyanobacteria and its derived pollutant MCs have become a global environmental problem. At present, there are relatively mature environmental exposure levels and toxic effects of MCs, in the future prevention and control of MCs pollution, the following points need to be paid attention to: (1) Monitoring the content of MCs in lakes and rivers across the country, and it is necessary to control the water beyond the specified concentration of MCs in time. (2) Strengthening the accumulation and transmission of MCs at all levels of trophic levels in the aquatic ecological food chain. Exploring the mechanisms and pathways of MCs in natural degradation, chemical degradation, and biodegradation. (3) In view of the current analysis of the transmission and transport mechanism of MCs in the process of cyanobacteria resource utilization, it is necessary to further study the exposure of MCs in the process of resource utilization, which is of great practical significance for predicting environmental risks. (4) MCs do not appear alone in the environment. The comprehensive toxicity formed by the combination of organic and inorganic pollutants in the environment and algal toxins may cause greater harm. Therefore, further research on the combined pollutants of different types of MCs can predict and reduce environmental risks. (5) While detecting the content of MCs, it is necessary to find relevant molecular markers that indicate the pollution of cyanobacteria blooms, which is conducive to studying the toxic mechanism of MCs and further judging the ecological safety of lakes in the early stage.

## Data Availability

Not applicable.
